# Caregiver perspectives on participation in pediatric neuroimaging research: insights from qualitative focus groups

**DOI:** 10.3389/fnins.2026.1963995

**Published:** 2026-10-14

**Authors:** Sydney Olson, Carly Reineri Thellman, Brooke Demetri, Brittney Sholar, Sarah Shultz

**Affiliations:** 1 Marcus Autism Center, Children's Healthcare of Atlanta, Atlanta, GA, United States; 2 Department of Pediatrics, Emory University School of Medicine, Atlanta, GA, United States

**Keywords:** caregiver perspectives, family-centered research, infant MRI, natural sleep neuroimaging, participant experience, qualitative research

## Abstract

Infant functional and structural magnetic resonance imaging (MRI) is increasingly used in research to identify early emerging biomarkers of neurodevelopmental conditions. Although these methods hold promise for advancing discovery, pediatric MRI research protocols, which often require infants to be scanned during natural sleep, can present a range of challenges for infants and their caregivers. Yet little is known about caregivers’ experiences participating in non-clinically indicated MRI studies with their infants. This study explored caregivers’ motivations for participating in infant MRI research, their experiences during participation, and their suggestions for improving infant MRI protocols. Three semi-structured focus groups were conducted with caregivers of 0-6-month-old infants who participated in a longitudinal MRI study. Thematic analysis revealed that caregivers were primarily motivated by contributing to science and helping other families. Many experienced performance anxieties while attempting to help their infants fall asleep for a scan, but supportive communication from research staff helped reduce these concerns. Caregivers recommended flexible scheduling, clearer procedural expectations, and pre-visit materials to improve preparedness. These findings underscore the importance of family-centered communication in infant neuroimaging protocols.

## 1 Introduction

Magnetic resonance imaging (MRI) is widely used to characterize infant brain organization and to investigate typical and atypical developmental trajectories, providing insights into the neural mechanisms underlying neurodevelopmental conditions (e.g., Graham et al., 2015; Banihashemi et al., 2026). Over the past decade, infant neuroimaging research has expanded substantially, owing in part to large-scale funded projects such as the Baby Connectome Project, Developing Human Connectome Project, and HEALthy Brain and Child Development Study (HBCD) (Howell et al., 2019; Edwards et al., 2022; Volkow et al., 2024). This work has generated foundational insights into early brain development, including the emergence and maturation of functional brain networks during infancy (e.g., Gilmore et al., 2018), the relationship between early brain development and later cognitive and developmental outcomes (e.g., Emerson et al., 2017; Girault et al., 2019), and the influence of environmental factors and early life experiences on neurodevelopmental trajectories (e.g., Tooley et al., 2021; Triplett et al., 2022).

Despite the substantial scientific value of infant MRI research, acquiring high-quality neuroimaging data from infant populations presents unique challenges (Spann et al., 2023). MRI scans are highly sensitive to motion, and although sedation is often used for clinically indicated MRIs, it raises ethical concerns in the context of non-clinically indicated research scans. To mitigate movement and increase infant comfort, researchers typically perform scans while infants are sleeping naturally, which introduces its own set of logistical and procedural challenges. These challenges include difficulties putting an infant to sleep in an unfamiliar environment, the significant time and caregiver involvement required during lengthy MRI visits, potential disruptions to infants’ sleep schedules, and caregiver concerns or anxiety about having their baby complete a clinically unnecessary MRI (Raschle et al., 2012a; Turesky et al., 2021).

Collectively, these challenges have important implications for caregiver willingness and ability to participate in repeated research visits, directly influencing not only retention and the overall success of longitudinal infant neuroimaging studies but also the quality of families’ research experiences. Consistent with these challenges, pediatric MRI studies report relatively moderate success rates, averaging approximately 70% across birth through early childhood (reviewed in Turesky et al., 2021) but declining to roughly 50–60% during infancy and toddlerhood (Knickmeyer et al., 2008; Raschle et al., 2012a). Longitudinal developmental studies are further affected by participant attrition, which can introduce bias and reduce statistical power, with retention varying according to demographic, psychosocial, and study-related factors (Turesky et al., 2021; Teixeira et al., 2021). Additional work suggests that barriers to participation in pediatric research include concerns about child safety, logistical burdens, historical mistrust of research, and stress associated with study participation, all of which can negatively affect enrollment and continued participation (Nathe et al., 2023). Attending to caregivers’ experiences may be particularly important during early parenthood, a period often characterized by heightened stress and vulnerability, especially for first-time caregivers (Turesky et al., 2021).

Large-scale longitudinal studies such as HBCD have emphasized the importance of participant engagement and family-centered practices for successful recruitment and retention of families with infants (Harden et al., 2024; Spann et al., 2023). Likewise, a survey of infant neuroimaging laboratories highlighted the extensive efforts that research teams undertake to facilitate infant sleep, reduce participant burden, and maximize successful data acquisition (Hendrix and Thomason, 2022). Together, these studies underscore the field’s recognition that caregiver engagement is central to successful infant neuroimaging research. Yet an important gap remains: relatively little is known about caregivers’ lived experiences of participating in non-clinically indicated infant MRI studies. Existing work has primarily examined caregivers’ MRI knowledge, research literacy, willingness to participate in infant MRI research, and strategies for reducing barriers to enrollment (Kohlasch et al., 2021), with comparatively little work exploring caregivers’ motivations for participation, their lived experiences throughout the research process, and their recommendations for improving infant MRI research based on their firsthand experiences.

To address this gap, we conducted qualitative focus groups with caregivers whose infants participated in a longitudinal MRI study of early brain development, including infants at both low and elevated likelihood for autism spectrum disorder. Focus groups explored caregivers’ perspectives on: (1) their motivations for participation; (2) their experiences during MRI participation; and (3) suggestions for improving study procedures. Insights from these discussions revealed aspects of the MRI experience that may not be readily apparent from investigator perspectives or traditional measures of recruitment, retention, and scan success, informing revisions to our infant neuroimaging research protocols and practical recommendations for investigators seeking to develop more family-centered infant neuroimaging research practices.

## 2 Methods

### 2.1 Parent study

All focus group caregivers participated in a larger longitudinal study of infants at low and elevated familial genetic likelihood for autism spectrum disorder conducted at the Marcus Autism Center. The broader study aimed to improve early detection and support for infants later diagnosed with autism. Infant MRI was one component of a comprehensive research protocol spanning birth to 30 months of age, which also included longitudinal eye-tracking and vocal recordings, standardized assessments of language, social communication, cognitive, and motor development, and diagnostic assessments for autism at 21 and 30 months. At enrollment, caregivers could elect whether or not to participate in the MRI component; MRI participation was not required for participation in the other components of the longitudinal study.

### 2.2 MRI protocol

MRI scans were conducted at the Emory Center for Systems Imaging using a 3 T Siemens Tim Trio scanner with a 32-channel head coil. Participants were healthy infants undergoing MRI solely for research purposes; scans were not clinically indicated. During the informed consent process, caregivers were explicitly informed that scans were conducted for research purposes only, and were not intended to identify any clinical conditions. Families were also informed of the study’s incidental findings policy. If the MRI technologist observed something of potential concern, the images would be referred to a pediatric neuroradiologist for review, and the family would be contacted if clinical follow-up was recommended. However, caregivers were advised that research staff were not trained to systematically identify clinical abnormalities and that the research sequences differed from those used for clinical evaluation; consequently, the absence of contact should not be interpreted as indicating the absence of a clinical finding.

To minimize motion and maximize scan quality, all scans were conducted during natural sleep. MRI data were collected at up to three time points between birth and 6 months of age to characterize trajectories of structural and functional brain development during early infancy. Sequences included T1-weighted, T2-weighted, diffusion, and resting-state fMRI acquisitions. T1-weighted images were acquired at 0.8 mm isotropic resolution (TR = 2,400 ms, TE = 2.22 ms); T2-weighted images at 1 mm isotropic resolution (TR = 3,200 ms, TE = 561 ms); resting-state fMRI at 2.5 mm isotropic resolution (TR = 720 ms, TE = 33 ms, multiband acceleration factor = 6); and diffusion images at 2 mm isotropic resolution (TR = 6,200 ms, TE = 74 ms, multiband acceleration factor = 2). Total scan time was approximately 30 min.

To determine eligibility for MRI participation, caregivers completed an MRI safety questionnaire assessing contraindications for MRI. Because scans were conducted during natural sleep, caregivers also completed a brief questionnaire regarding their infant’s typical sleep patterns. MRI visits were scheduled during regular business hours and aligned as closely as possible with the infant’s usual naptime.

Upon arrival, families were escorted to a family-friendly preparation room, equipped with an infant changing table and supplies (e.g., diapers, pacifiers, wipes, bottle warmer). The MRI safety questionnaire was repeated to confirm no new contraindications, and responses were reviewed by the MRI technician. Study staff described MRI procedures to families, conducted metal screening to ensure the removal of all ferromagnetic items, and secured personal items in lockers. Infants were changed into MRI-safe clothing if needed. Caregivers were then given a tour of the MRI suite and a more detailed overview of study procedures to increase comfort and familiarity.

Caregivers were invited to help their infant fall asleep using their preferred soothing methods (e.g., feeding or rocking), with research staff available to help if requested. An MRI-safe rocking chair was available in the scanner room, and staff stepped into the control room to provide privacy and reduce distractions while caregivers settled their infants. Caregivers were instructed to gently knock on the door to notify staff once the infant had fallen asleep or if assistance was needed. If staff did not receive communication from the caregiver after approximately 30 min, they quietly re-entered the room to check whether support was needed and whether caregivers wished to continue or conclude the visit.

Once asleep, infants were positioned in a padded, MRI-safe infant bed placed on the scanner bed. Custom infant sound-attenuating headphones were fitted and secured with foam padding. The headphones were equipped with in-ear microphones to record sound levels reaching infants’ ears throughout the scan. Sound was further reduced by a custom acoustic hood placed within the scanner bore. Together, the headphones and acoustic hood reduced sound levels to below 80 dBA for all sequences. A camera mounted to the head coil provided experimenters and caregivers with a clear view of the infant’s face throughout the scan.

Prior to scan start, white noise, gradually increasing in volume, was played through the headphones to mask the onset and offset of scan sequences. Caregivers were permitted to remain in the MRI suite throughout the scan. A trained experimenter remained present to monitor the live camera feed of the infant, while a second experimenter in the control room monitored real-time sound levels reaching the infant’s ears. Heart rate and respiration were not monitored during scanning. The sequence was stopped if the infant awoke or if sound levels exceeded established thresholds. If the infant awoke, caregivers could choose whether to attempt resettling or conclude the visit. Families received $50 per MRI visit regardless of whether usable scan data was obtained.

### 2.3 Focus group participants

Participants in the present study were 10 caregivers of 11 infants (aged approximately 1.0–6.6 months at the time of MRI participation), including one caregiver of twins, who were enrolled in the parent study at the Marcus Autism Center. Caregivers were recruited using purposive sampling from families who had completed the infant MRI protocol within the prior 6 months to ensure recency and familiarity with their experiences. Recruitment also aimed to achieve representation across several potentially important dimensions, including scan success (i.e., inclusion of caregivers whose infants successfully completed a scan and those whose infants did not), socioeconomic status (indexed by household income and maternal education), and race. Caregivers meeting these criteria were identified from study records and were invited by email to participate in the focus group. The resulting sample was broadly representative of the racial and socioeconomic composition of the larger study cohort. Although the focus group interview guide was not designed to probe for differences as a function of familial genetic likelihood status for autism, we recruited equal numbers of caregivers of infants at low and elevated likelihood for autism, consistent with the composition of the broader longitudinal cohort and to allow for the possibility that experiences may differ across these groups. Recruitment continued until code saturation was reached, as described below.

Three semi-structured focus groups were conducted (2–5 caregivers per group) to elicit caregiver perspectives on (1) their motivations for participation; (2) their experiences during MRI participation; and (3) suggestions for improving study procedures. All caregivers provided informed consent and were compensated for their time. Caregiver and infant characteristics are summarized in Table 1.

**Table 1:** Caregiver characteristics and infant MRI completion.

Age	Relationship	Education	Race	Household Income	ASD Likelihood	MRI 1	MRI 2	MRI 3
35	Biological mother	Professional degree (MD, JD, PhD)	White	$80,001–$100,000	LL	Partially completed	Sleep not achieved	Sleep not achieved
32	Biological mother	Master’s degree	White	$125,001–$150,000	LL	Partially completed	Completed	Completed
34	Biological mother	Professional degree (MD, JD, PhD)	White	$100,001–$125,000	EL	Completed	Partially completed	Completed
45	Biological father	College degree	More than One Race (Asian and White)	$125,001–$150,000	EL	Completed	Sleep not achieved	Did not attend
20	Biological mother	Courses toward college	White	$20,001–$40,000	LL	Partially completed	Completed	Sleep not achieved
42	Biological mother	Courses toward college	Black or African American	$20,001–$40,000	EL	Sleep not achieved	Sleep not achieved	Sleep not achieved
25	Biological mother	College degree	More than One Race (Black and White)	$40,001–$60,000	EL	Partially completed	Partially completed	Partially completed
29	Biological mother	College degree	White	$125,001–$150,000	LL	Partially completed	Completed	Canceled by study team
36	Biological mother	Master’s degree	White	$100,001–$125,000	EL	Completed	Completed	Completed (twin A); sleep not achieved (twin B)^a^
23	Biological mother	College degree	White	$40,001–$60,000	LL	Completed	Completed	Completed

EL = elevated likelihood for ASD; LL = low likelihood for ASD. All caregivers identified as not Hispanic or Latino. MRI visit completion status categories were defined as follows: ‘Completed’ = infant slept for entire scan session; ‘Partially Completed’ = some MRI data acquired but not fully completed due to infant awakening; ‘Sleep Not Achieved’ = MRI visit could not be initiated because the infant did not fall asleep; ‘Did Not Attend’ = participant did not present for scheduled visit; “Canceled by study team” = visit canceled due to study-related factors. ^a^Caregiver participated with twins; MRI Visit 3 outcomes differed by twin.

### 2.4 Focus group design and procedure

Focus groups were conducted using a semi-structured interview guide developed by the research team, which included open-ended questions addressing motivations for participation, experiences during MRI visits, perceptions of protocol acceptability, and suggestions for improvement (see Appendix A). Open-ended prompts were used to initiate discussion, while follow-up probes were used to clarify perspectives and encourage elaboration. Interviews were conducted by the study principal investigator (SS). SS was an investigator on the infant MRI study and has a professional interest in understanding and improving families’ experience with infant MRI research. SS had no interaction with participants during their recruitment, enrollment, or study visits and had no contact with participants before or after the focus group. Her lack of involvement in participants’ study visits was intended to allow caregivers to share their experiences more freely without concern that they were reporting to study staff involved in their prior research interactions. At the beginning of each focus group, SS introduced herself as an investigator on the MRI study. Caregivers were informed that the discussion was intended to focus specifically on their experiences with the MRI component of the study rather than the broader parent study. Each focus group lasted approximately 60–90 min and was conducted in person in a private conference room. All sessions were audio-recorded and transcribed by a professional human transcriptionist after the removal of identifying information. Any unclear passages within the transcript were checked against original audio recordings to verify wording and context.

### 2.5 Coding and analysis

Following each focus group, two investigators (CR and BS) independently reviewed the transcript and identified emerging codes and thematic content, which they compared and organized into a developing codebook. Following each successive focus group, the investigators assessed whether new thematic content emerged that required additions to the codebook. When the third focus group yielded no new thematic content requiring additions, CR, BS, and SS jointly determined that code saturation had been reached and recruitment was concluded (Hennink et al., 2017).

Following completion of data collection, CR and BS reviewed and refined coding across all three transcripts. Codes were compared, discussed, and organized into broader thematic categories, resulting in a final codebook encompassing three key areas: caregiver motivations for participation, caregiver experiences during MRI visits, and caregiver suggestions for improving the MRI research experience (sample codebook presented in Appendix B). Qualitative coding and analysis were conducted without the use of qualitative analysis software.

Two additional investigators (BD and SO) then independently applied the finalized codebook to all transcripts. The coded data were subsequently reviewed and organized into themes and subthemes, and representative quotations were selected to illustrate each theme.

## 3 Results

### Aim 1. motivations for participation

An overview of the major findings and associated thematic codes for Aim 1 is provided in Table 2.

**Table 2:** Summary of qualitative findings and associated codes for study aim 1.

Major finding	Associated codes	Brief description
1a. Contributing to science and helping other families	Help science; contributing to research; helping another family	Caregivers described contributing to scientific research and potentially helping other children and families as important motivations for participation.
1b. Developmental monitoring as a motivation for participation	Benefit child early assessments; information	Caregivers valued opportunities to learn about and monitor their infant’s development through participation in the broader longitudinal study.
1c. Appreciation for keepsake images of their infant’s developing brain	Look at child’s brain; curiosity; souvenir brain picture “brain diplomas.”	Caregivers described curiosity about seeing their infant’s brain and valued receiving non-clinical brain images as a keepsake of participation.

### 1a. Contributing to science and helping other families

Caregivers in all three focus groups expressed that a strong motivator for joining the study was a desire to support scientific advancement and the well-being of children.

“I think it’s nice too when people who aren’t science people and do not have experience with this can feel, I mean I know we keep saying it, but to feel a part of something bigger is actually a really big benefit and it’s an intangible benefit.” (Caregiver, Focus Group One).

### 1b. Developmental monitoring as a motivation for participation

Although focus group questions centered on the MRI component, caregivers also described motivations that pertained to participation in the broader parent study rather than MRI specifically. In particular, many caregivers valued the developmental information and monitoring they received through the parent study and appreciated the opportunity to take proactive steps in supporting their infant’s development. These perceived benefits arose from participation in the broader developmental study and should not be interpreted as benefits of the MRI component itself.

“I think it [the study] came at a perfect time for me because my older son got diagnosed with autism when he was four and I was pregnant. They’re [care team] telling me about his diagnosis, they are also saying, ‘We see that you might be a good fit for these studies.’ It probably wasn’t the perfect time because I already had a lot on my plate, but we had waited since February to get him in [for an evaluation] to November to get in at all. My husband was like this gets him on the list regardless if we see anything—he will be tested, and we will know. So it did seem like even though at the time, you know you have a higher risk and everything, it’s also like well we are doing everything we possibly could from the get go, so it did make you feel better even though you were not necessarily doing anything about it, but at least he’ll be well examined by the top people, so it did sort of make it feel like you were doing something.” (Caregiver, Focus Group One).

### 1c. Appreciation for keepsake images of their infant’s developing brain

As a part of participation in the longitudinal infant MRI study, caregivers received structural images of their infant’s brain following each scan, referred to as “brain diplomas,” as a token of appreciation for participation. While the structural images were non-clinical in nature, many caregivers expressed appreciation for receiving a memento of their infant’s developing brain.

“I was excited when they said we would get a copy of the pictures [brain images].pretty cool to have that for our records.” (Caregiver, Focus Group Two).

### Aim 2. Caregiver experiences throughout study participation

An overview of the major findings and associated thematic codes for Aim 2 is provided in Table 3.

**Table 3:** Summary of qualitative findings and associated codes for study aim 2.

Major finding	Associated codes	Brief description
2a. Initial reactions to and preparing for MRI participation	Easy pre-scan experience; difficult pre-scan experience; anxiety the night before scan; first time mom feeling of protection; positive experience in keeping packets of info at home/plenty of info; positive consent experience; anxiety in consent	Initial reactions ranged from comfort with participation to concerns about MRI safety and procedures, with detailed information and opportunities to ask questions supporting preparation and confidence prior to the visit.
2b. Scheduling, travel, and logistical barriers to participation	Child would fall asleep on way to scan; accommodating scheduling experience; would have rather more accommodating scheduling	Scheduling and travel presented practical barriers, including work and family responsibilities, traffic and long commutes, limited appointment flexibility, and travel interfering with infant sleep.
2c. Infant sleep challenges and caregiver performance anxiety	Unpredictable nap schedule; stress about baby not falling asleep/performance anxiety; child did not sleep; child woke up; knew from beginning child would not sleep; difficult time getting child to sleep; difficult time putting child on bed; good experience with white noise; performance anxiety overall; said nothing could fix performance anxiety	Unpredictable infant sleep patterns complicated visit scheduling and scan preparation, while difficulties getting infants to sleep or remain asleep contributed to caregiver anxiety and a sense of responsibility for scan success.
2d. Communication and reassurance during MRI visits	Felt reassured throughout process; difficulty with communication that child was asleep; unsure of how to communicate with research coordinator; fine experience communicating with research coordinator; liked camera of baby’s face; stressed watching baby on screen; afraid baby would wake up; afraid baby would move; communicate with parent before pulling out of machine; more support for parent throughout process	Supportive communication, reassurance, and flexibility during MRI visits reduced pressure and helped families feel supported, while responses to viewing infants on the live camera feed varied from reassurance to increased anxiety.
2e. Growing familiarity with MRI experience across visits	Fine experience in scan room; concerning feelings about machine; anxiety decreased with subsequent visits	Initial comfort with the MRI environment varied, while increasing familiarity with study procedures and staff generally reduced anxiety

### 2a. Initial reactions to and preparation for MRI participation

When caregivers reflected on their initial decision to participate in the MRI study, they described a range of reactions. Some reported questions or concerns related to MRI safety, unfamiliarity with the procedure, or the use of medical equipment, whereas others recalled feeling comfortable with participation from the outset. Across focus groups, caregivers emphasized that receiving detailed information and having opportunities to ask questions helped them feel informed and confident in their decision to participate.

“My husband said no immediately. I was on the fence. I think like, anything involving any kind of medical equipment and stuff, he was very leery of it. But then they immediately handed us the information packet, and we were able to go through it and ask [the researcher] any questions, we felt comfortable trying it, and it was fine.” (Caregiver, Focus Group Three).

“I can see how MRI would sound very intimidating and like how a mom, especially a new mom, would not want to go through that process, but I do not know. Like I feel like if I wasn’t as informed as I was going into it, I feel like I either would not have done it, or I would have hesitated more.” (Caregiver, Focus Group Three).

Although caregivers generally felt prepared before their infant’s MRI visit, one caregiver described feeling nervous the night before the appointment. Despite receiving detailed information about scan safety and procedures, this caregiver attributed her feelings to lingering postpartum sensitivity and a strong sense of protectiveness towards her infant.

“And the night before, even though I knew how safe it was and I had the expert go and tell me in detail, you know what it is going to be like, but the night before, I got really nervous. I do not know, it was a little postpartum leftover—I felt protective.” (Caregiver, Focus Group One).

### 2b. Scheduling, travel, and logistical barriers to participation

Scheduling infant MRI visits required balancing family preferences with the practical constraints of the imaging center’s weekday operating hours and scanner availability. Within the focus groups, caregivers described their experiences with barriers such as traffic, long commutes, uncertainty about how flexible time slots could be, and limited availability during non-working hours.

“The difficulty for us was work and scheduling—figuring out who could take the time off. It did not seem like you guys do any Saturday appointments, but if that’s an option at all, like, you know, if the baby’s just not cooperating and they cannot keep coming back, that could be pretty helpful option for where both parents are working.” (Caregiver, Focus Group Two).

“I think something that had to be morning, but then I was leaving like over an hour in advance because there was so much traffic. Like it was always during traffic, and it was like at that point [baby] could not sleep in the car very well, and it was like a horrible drive.” (Caregiver, Focus Group Three).

### 2c. Infant sleep challenges and caregiver performance anxiety

Across all focus groups, caregivers reported that it was challenging to get their baby to sleep during the scheduled MRI visits, primarily due to the unpredictable and evolving nature of sleep patterns in early infancy. Although visits were scheduled to align with each infant’s typical nap time, many caregivers expressed uncertainty about how to select an appropriate time, especially during the earliest months when sleep patterns were still forming. To support scheduling and tailoring procedures to family needs, families completed a nap-time questionnaire prior to visits; however, caregivers expressed mixed views regarding its usefulness. Some participants noted that the questionnaire was difficult to answer for infants without a consistent nap schedule and, in some cases, increased anxiety rather than reducing it.

“That age is really hard to even predict [a nap schedule] a day before, much like a month before—pretty much impossible. It may be [useful] for most people, but I feel like for my kids, their nap times are so fluid.” (Caregiver, Focus Group Two).

“I don’t know. Parents just differ so much. What if baby’s co-sleeping, or there are so many different parenting styles. I can definitely see how knowing the nap time or schedule is helpful to the team, but I don’t know. I think that was the intimidating part of everything, rather than the actual MRI—I don’t know when he [baby] sleeps.” (Caregiver, Focus Group Three).

Challenges related to infant sleep continued once caregivers arrived for the MRI visit. Caregivers reflected on the process of transferring and positioning their sleeping infant on the scanner bed and preparing them for scanning once asleep. Across focus groups, caregivers described this stage as particularly delicate. One caregiver highlighted several factors as helpful for maintaining infant sleep during scan preparation, including a sleep-conducive environment, clear coordination with research staff, and minimizing disruptions during equipment placement.

“For us, it went well, but I feel like it was really dark, which helped, and I feel like the machine’s white noise for him (baby falls asleep with white noise) was good enough at mimicking what we do at home. It was definitely helpful. It’s also really helpful that there were three of us, so I was kind of doing something soothing, and they were very quickly doing the adjusting. I don’t know what you could do if it was going to be a twitchy day or not.” (Caregiver, Focus Group One).

These challenges surrounding infant sleep were accompanied by anxiety about whether infants would fall asleep or remain asleep long enough for useable data to be collected. Although research staff were available to assist, caregivers typically led the process of helping their infants fall asleep, while research staff remained in the control room for privacy. Several participants described internalizing a sense of responsibility for the success of the scan and expressed concern that they had let the research team down if their child was unable to sleep. In Focus Group One, one caregiver described this experience as “performance anxiety,” a term subsequently used by the moderator to explore similar experiences in Focus Group One and Two. Caregivers in Focus Group Two endorsed similar feelings when the term was raised. Although the term itself was not used in Focus Group Three, caregivers similarly described anxiety surrounding getting their infant to sleep and scan success.

“I kind of felt like I kind of let you guys down, like when she wouldn’t sleep, maybe I could have done something differently to help. I mean, we all got here for her to do this, then maybe I couldn’t do my part to get her to sleep. […] you can tell me all day long that it’s fine, but I kind of felt like I’m sorry.” (Caregiver, Focus Group Two).

“I think I had more like anxiety about getting him to sleep than the actual MRI machine.” (Caregiver, Focus Group Three).

### 2d. Communication and reassurance during MRI visits

Despite anxieties related to their baby falling or remaining asleep, caregivers across all three focus groups described the overall MRI visit as supportive and reassuring. Many reported that the research team’s communication style played a significant role in their sense of reassurance. Caregivers emphasized the importance of being reminded that their participation was valuable regardless of sleep outcome and appreciated having the option to stop the scan at any time, reschedule visits as needed, and ask questions throughout the process.

Caregivers also described feeling reassured when study procedures remained flexible and responsive to their child’s needs and cues. One caregiver in Focus Group One emphasized that reducing pressure and adapting participation to their infant’s needs helped create a more positive experience. Knowing that their child could guide the pace of the visit contributed to a sense of support and reduced concern about whether or not their baby would fall asleep.

“I had never been in charge of anyone really besides myself, and here I am deciding whether or not to put this tiny human in a machine that seems very intimidating, and I was exhausted, so I think those were definite factors, but I did feel supported. I felt overly coddled in a way I think was very helpful. I was told over and over again that we could stop at any time, over and over again that I could change my mind, over and over again, that if I had any questions, I could talk to the technician. I found all that really helpful and reassuring.” (Caregiver, Focus Group One).

“I think I felt more of that [reassurance] after the appointment and after he didn’t fall asleep [the researcher] was very reassuring of this happens all the time, the babies, like a ton of our babies don’t fall asleep and we can always try again and it’s no big deal and I was like oh, okay, we can come back and we can still make this work and we can still do the next month, then I felt a lot more reassured.” (Caregiver, Focus Group Three).

Caregivers also discussed the live camera feed (an MRI-safe camera affixed to the head coil, allowing caregivers to see a live feed of their infant as they slept in the scanner) as a way of remaining connected to their infant during scanning. Several caregivers found being able to see their infant was reassuring, although one caregiver reported that watching the feed increased anxiety when the infant moved or appeared likely to wake.

“I liked the camera and having a researcher in the room watching the camera as well. I could see their communication, and I could also weigh in.” (Caregiver, Focus Group One).

“It stressed me out a little bit. I could say you want them to be looking at it, but then also just not wanting him to wake up and scream and be upset. He didn’t wake up, and when he did open his eyes, they were done, but he opened his eyes and just sort of looked around like ‘okay’. So, he didn’t really care, but especially the first time, I think I was a little worried that he’d wake up and be like where am I?” (Caregiver, Focus Group One).

### 2e. Growing familiarity with MRI experience across visits

Caregivers described varying levels of familiarity with the MRI environment at their initial visit. One caregiver remarked on the striking visual contrast between the large MRI scanner and their small infant, which made the environment feel intimidating at first glance. Another caregiver expressed minimal concern due to prior experience with MRI. One caregiver, despite having no prior exposure to an MRI scanner, did not find the environment intimidating.

“It’s a big machine and you have this like itty bitty thing [baby].” (Caregiver, Focus Group Three).

Across subsequent visits, caregivers described increasingly positive experiences, noting that familiarity with the study procedures and staff reduced anxiety and enhanced comfort over time. Seeing familiar staff members, knowing what to expect, and being welcomed by name created a sense of community.

“I felt like you knew what to expect a lot better… like the subsequent ones felt relatively short in comparison, but yeah, I mean because I knew where to go and all that, like it went a whole lot smoother and everything was all right.” (Caregiver, Focus Group One).

“People involved got to know him all around from just checking in, remembering his name, and saying how he’s grown, and that’s just a good feeling. It makes you feel like you’re a part of the community of people trying to do research.” (Caregiver, Focus Group One).

### Aim 3. Caregiver recommendations for improving the infant MRI research experience

An overview of the major findings and associated thematic codes for Aim 3 is provided in Table 4. Caregiver-identified challenges and recommendations arising from Aim 3, along with their potential implications for infant MRI protocols, are summarized in Table 5.

**Table 4:** Summary of qualitative findings and associated codes for study aim 3.

Major finding	Associated codes	Brief description
3a. Preparing families for MRI	Short video of MRI study process; overview context of study schedules at first visit to know what to expect; call the day before; text the day before; email day before	Providing concise information about the MRI experience before the visit and reminders through families’ preferred communication methods may help set expectations and support preparation.
3b. Improving communication throughout the MRI visit	Better communication that baby is asleep; button; communicate through window; clock in scan room; communicate time parameters with parent throughout; explain starting and stopping of scanner	Clearer and less disruptive communication during MRI visits, including ways to signal when an infant is asleep, explanations of scan sequences, and access to information on time, may reduce uncertainty and disruptions during scanning.
3c. Supporting infant comfort and natural sleep	Lower lights in family/control room; white noise/music	A calm, sleep-conducive environment that incorporates familiar sensory supports, such as low lighting and white noise, may help infants settle naturally and improve family preparedness for visit.
3d. Reducing caregiver pressure and setting expectations for scan success	Reassure parent that it’s okay if baby does not sleep; do not be pushy about baby falling asleep; continue the focus to be on child “baby is boss”	Setting realistic expectations for scan success and emphasizing that participation remains valuable even when an infant does not sleep may help reduce pressure while keeping the infant’s needs central to the visit.
3e. Adapting scan procedures to infant sleep and behavior	Less manipulation of baby once on bed/asleep; keep going if baby is still asleep; keep baby in scanner, if baby will fall back asleep	Allowing time for infants who stir to resettle and extending scanning when infants remain asleep may better align scan procedures with individual infant sleep and behavior.
3f. Supporting caregivers in explaining and sharing study participation	More study information for friends/family not in research; better name of study to explain to peers	Accessible, shareable study information may help caregivers explain participation to others and address potential misunderstandings about infant MRI research.

**Table 5:** Caregiver identified challenges, recommendations, and potential implications for infant MRI protocols.

Caregiver identified challenge	Caregiver recommendations	Potential implications for infant MRI protocols
Difficulty anticipating the MRI experience and processing information during the visit	Provide a brief pre-visit video (about one to two minutes) showing the MRI environment and explaining key procedures, safety information, and expectations	Providing pre-visit orientation that allows caregivers to see the MRI environment and procedures may help bridge the gap between receiving information and experiencing the MRI firsthand, while reducing the amount of new information caregivers must process before the demands of the MRI visit.
Notifying research staff that the infant was asleep risked disrupting sleep	Provide a quiet method for caregivers to notify staff when their infant has fallen asleep or if they need assistance, such as a button or visual signal	Communication procedures should minimize disruptions during sensitive transitions.
Uncertainty about scan timing, progress, pauses, and restarts	Explain expected scan sequences and pauses in advance and provide a visible clock or periodic updates about elapsed time and scan progress	Incorporating clear time cues and scan progress updates may reduce uncertainty for caregivers during natural sleep MRI visits.
Difficulty settling and maintaining infant sleep in the MRI environment	Support a calming, sleep conducive environment through strategies such as low lighting and white noise, and communicate available sleep supports to caregivers prior to the visit	Natural-sleep MRI protocols should account for individual differences in how infants fall and remain asleep and allow flexibility in the strategies families use to support sleep.
Caregiver performance anxiety and perceived responsibility for scan success	Normalize unsuccessful sleep or scan attempts, emphasize that participation remains valuable even when usable data are not obtained, and clearly communicate expectations for scan success and options for reattempting the scan	Clear expectations and reassurance may help address caregiver performance anxiety, although some caregivers may continue to experience a sense of responsibility for the scan success despite reassurance from the research team.
Scans were sometimes ended when caregivers felt their infant might resettle or continue sleeping	Allow infants who stir or briefly wake time to resettle before ending the scan, and when feasible, continue scanning while an infant remains asleep	Decisions about when to end scanning may benefit from greater flexibility in response to individual infants’ sleep and behavior, potentially maximizing opportunities for data collection.
Individual differences in preferences for pre-visit communication	Offer flexibility in how families receive pre-visit reminders and information, including phone, email, or text Allowing families to select a preferred communication method may make pre-visit outreach more responsive to individual family needs and circumstances	Allowing families to select a preferred communication method may make pre-visit outreach more responsive to individual family needs and circumstances.

Caregiver-identified challenges and recommendations are summarized from the findings in Aim 3. Potential implications reflect the authors’ interpretation of how these findings may inform the design and implementation of natural-sleep infant MRI protocols.

### 3a. Preparing families for the MRI visit

Caregivers described receiving information about the MRI procedures during the consent and enrollment process; however, more detailed explanations of the scanning environment were often provided during the study visit itself, sometimes while they were simultaneously trying to feed or rock their baby to sleep. While participants appreciated receiving this information, several suggested that providing a brief overview prior to the visit would help set expectations and reduce stress. A few caregivers recommended a short video (around 1–2 min in length) introducing the scanner environment and key procedures. Providing this information beforehand may help reduce cognitive load during the visit. One caregiver in Focus Group Two noted that a pre-recorded video with bullet points could efficiently convey safety information and logistical expectations.

“A video would be helpful because then you have something visual, and you can kind of see how it works—especially if you’ve never had an MRI—it would be helpful.” (Caregiver, Focus Group Three).

When asked about how pre-visit study information and reminders could best be communicated, caregivers across all three focus groups emphasized the value of receiving a reminder shortly before their MRI visit. However, the preferred method of outreach varied by individual. Some appreciated a phone call a day or two in advance, while others indicated email or text would be more effective, especially in busy moments when a phone call might feel intrusive. These mixed preferences highlight the importance of offering flexible communication options to ensure families feel prepared without being overwhelmed.

“Maybe a call the day before or two days before to remind me.” (Caregiver, Focus Group One).

“So I would definitely say email would be good because when you’re nursing a baby, or you are trying to feed and you’ve got this going on, that going on, if you’re trying to call me on the phone and I’m in the middle of that or even if they’re laying down and this is my one chance to breathe that day, I don’t want to talk on the phone.” (Caregiver, Focus Group Three).

### 3b. Improving communication throughout the MRI visit

Caregivers in Focus Groups One and Three described the process of notifying staff when their infant had fallen asleep—typically by knocking on the MRI room door—as disruptive to infant sleep. Caregivers suggested a button or visual cue they could initiate as a quieter alternative to communicate readiness while minimizing disturbances during a key moment of the visit.

“The knocking, and I’d see her jerk a little, so I don’t know if they can just—because they got that glass window right there. Maybe somebody could just stand there, and then I could just signal instead of knocking on the door.” (Caregiver, Focus Group Three).

“Maybe if there was a button to push or something just to get them back in, I just don’t want to feel like once I get my child to sleep, what am I supposed to do?” (Caregiver, Focus Group Three).

Once scanning began, caregivers also recommended clearer communication about when scan sequences would start and stop, as some mistook temporary silence as the end of the procedure.

“Maybe you could explain that it’s going to happen up front like this, and this is what you’re going to hear. We’re doing this many series.” (Caregiver, Focus Group One).

“I do not think it was jarring for me, but I was like ‘we are done’, and then it started over again, then I was like, “Oh, we are not done.” (Caregiver, Focus Group Two).

Caregivers also identified knowing how much time had passed as an important aspect of communication during the visit. Due to MRI safety procedures, caregivers were not allowed to bring in items such as a cell phone or watch inside the scanning room, which made it challenging to track the passage of time while settling their infant. As a result, caregivers across the focus groups suggested that a visible clock or periodic time updates from the research team would have helped ease anxiety and gain insight into when it might be appropriate to end the visit if the baby was not likely to fall asleep.

“Maybe like a clock or something in there so you can tell. My husband got out of there, and he’s like “I felt like we were in there for four hours” and it just—we were just sitting there thinking, “please, please sleep.” (Caregiver, Focus Group Two).

### 3c. Supporting infant comfort and natural sleep

Caregivers emphasized the importance of sensory elements that support a calm scan environment, particularly components that mimic the infant’s typical sleep settings. Across the focus groups, caregivers highlighted the value of playing white noise or low lighting to promote sleep and reduce disruptions during the scanning process, especially for infants who are light sleepers or have difficulty settling into a new environment. Caregivers noted that greater attention to communicating these available resources to support families to facilitate the natural sleep process may enhance preparedness and comfort prior to visit.

“The little headphones, I do not think it was made clear to me that the headphones.

Would have the white noise option, so maybe that needs to be said, just told to the parents because they were like oh, we can add that. I was like “Oh, we can add that. That would be good.” (Caregiver, Focus Group Three).

“I feel like it was really dark, which helped, and I feel like the machine’s white noise for him, like he falls asleep with white noise.” (Caregiver, Focus Group One).

### 3d. Reducing caregiver pressure and setting expectations for scan success

When caregivers were asked about how the research team could help alleviate performance anxiety related to infant sleep, they offered several suggestions for supporting families throughout the MRI process. Caregivers reiterated the importance of being reassured that many babies do not fall asleep and that their participation is valuable, regardless of whether or not data were collected. One caregiver shared that greater transparency could be helpful, for example, by being upfront about what constitutes adequate data collection and options for scan reattempts. Not all caregivers, however, felt that performance anxiety challenges could be fully addressed through changes to study procedures or communication. Another caregiver stated that no amount of reassurance could ease the internalized pressure, and the feeling of letting the team down was inevitable.

“I think I felt more of that [anxiety] after the appointment and after he didn’t fall asleep. The [researcher] was very reassuring that this happens all the time, tons of babies don’t fall asleep, and we can always try again. I was like, ‘Oh, we can come back… we can still make this work.’ Then I felt a lot more reassured. I think if I’d had that reassurance before, I would’ve felt more confident going into it and more relaxed in the process of getting him [baby] to sleep.” (Caregiver, Focus Group Three).

“I don’t think there was anything you could have said to me at least, because I was told it would be okay if she [baby] wouldn’t sleep, but for me, I felt like I let you down.” (Caregiver, Focus Group Two).

### 3e. Adapting scan procedures to infant sleep and behavior

Caregivers were able to monitor their baby during the scan via a live video feed. Several reflected on moments when their baby stirred or briefly opened their eyes. While research staff tended to interpret these signs as a cue to end the scan, caregivers disagreed with this approach. Instead, they suggested a more patient approach to pause and give the baby some time to settle rather than removing them immediately.

“I feel like they weren’t sure what would happen with the baby, similar to how you were saying, like the half-open eye sleeping, or the unsureness of how they sleep, or how they startle, or everything like that. I feel like when he opened his eyes a little bit, they were like we should like stop, get all the equipment off, take him out, and I was like just let him sit for a second and like don’t—just like don’t let him see you and then he went back to sleep.” (Caregiver, Focus Group Three).

Caregivers similarly expressed willingness to allow their infants to continue scanning beyond the allotted scan duration when their infant remained asleep. MRI sessions were scheduled in two-hour slots to allow time for infants to fall asleep, and for approximately 30–40 min of scanning. Total visit duration varied depending on how easily their infant fell and remained asleep. Caregivers’ willingness to continue scanning suggests that families may welcome flexibility in scan duration when aligned with the infant’s natural sleep period.

“I think I was shocked that he [baby] made it that long, but I think I guess I would have kept going until or for longer since he was still asleep. Like when he came out, I just let him sleep there.” (Caregiver, Focus Group Three).

### 3f. supporting caregivers in explaining and sharing study information

While caregivers in the focus groups generally felt well-informed by the research team, many expressed difficulties relaying study information to others, such as friends or family. One caregiver in Focus Group One described concerns about potential judgement when sharing details about the study with neighborhood groups, worrying that others might misunderstand the research as unsafe. This caregiver suggested the research team create informational materials with accessible and friendly language to help communicate the study to others. Other caregivers suggested that a study website could help to share study information with others.

“I’ve promoted it [the study] to play groups in our neighborhood and stuff like that, but I’m always a little afraid people are going to look into it like you’re experimenting on your child or whatever. I wish there was a little more information to destigmatize the idea of it being like an experiment on your child. Once the parents are in the study, they could have a folder to have a conversation with a friend they think might be a good fit. You can have language that is easily accessible, not intimidating, and friendly, so you could help sell or explain it to other people.” (Caregiver, Focus Group One).

“So maybe that’s where it would be good to have a website but linked for this study because you can easily just text a website to somebody or send a link, you know, that’s super easy.” (Caregiver, Focus Group Three).

## 4 Discussion

In this study, we conducted three semi-structured qualitative focus groups with caregivers whose infants participated in a longitudinal infant MRI study to better understand their motivations for participation, experiences during study visits, and recommendations for improving study protocols. Caregivers were strongly motivated by contributing to science and helping other children and families and generally described their experiences as positive and supportive. At the same time, participation involved notable challenges, particularly surrounding infant sleep and caregivers’ sense of responsibility for scan success. Caregivers also provided actionable, experience-based recommendations to improve study procedures and communication. These findings underscore the importance of centering caregiver perspectives in the design and implementation of infant neuroimaging research.

### 4.1 Caregivers are motivated to further science and help others

Caregivers across all focus groups described a desire to contribute to scientific knowledge and to help other children and families as a primary motivation for participation. These findings are consistent with the literature on pediatric clinically indicated studies, where altruism emerged as an important motivator for caregiver participation in research (Weiss et al., 2024), and highlight the importance of clearly communicating research goals, potential scientific impact, and study findings to participants, as understanding how their contributions advance knowledge may help sustain engagement and reinforce the sense of purpose that motivates participation.

### 4.2 Caregiver concerns beyond MRI safety and burden

Prior to conducting the focus groups, we hypothesized that caregivers’ primary participation challenges might relate to MRI safety, the intimidating nature of the scanner environment, or the logistical burdens associated with participation. However, caregivers unexpectedly consistently identified uncertainty surrounding infant sleep as the primary challenge associated with participation in the research study. While some caregivers described initial questions regarding MRI safety, unfamiliarity with the scanner environment, or logistical challenges, these concerns were generally modest and did not emerge as a dominant feature of participation.

Instead, caregivers consistently identified uncertainty surrounding infant sleep as the most salient challenge. Concerns centered not on the MRI itself, but on whether their infant would fall asleep and remain asleep long enough to acquire the data needed for a successful scan. This finding was particularly notable, given that several aspects of our protocol were designed to address the concerns we had originally anticipated (i.e., concerns about the MRI itself and time burden). For example, we were initially reluctant to offer additional scan attempts or extended scan time out of concern that families would perceive these options as burdensome. In contrast, caregivers in the present study often viewed the opportunity for additional scan attempts as reassuring, particularly when communicated in advance. Likewise, procedures intended to minimize caregiver distress, such as promptly stopping a scan if an infant awoke, did not always align with caregivers’ priorities, given the importance they placed on successful data acquisition. These findings highlight how investigators’ assumptions about potential challenges may not always align with families’ experiences of participation.

### 4.3 Performance anxiety and perceived responsibility for scan success

Relatedly, caregivers in all focus groups frequently described feeling personally responsible for their infant’s inability to fall or remain asleep during the scan. Several caregivers described feeling as though they had let the research team down when their infant was unable to complete the protocol, despite repeated reassurance that unsuccessful scan attempts were expected and still valuable.

This finding expands our understanding of the factors that shape caregiver experiences in infant neuroimaging research. Previous work has described practical and logistical barriers, including travel demands, scheduling difficulties, and time commitment (Nathe et al., 2023; Turesky et al., 2021). While these challenges were also present in the current study, caregivers frequently described an additional emotional burden associated with participation. Many caregivers appeared to internalize responsibility for scan success, describing feelings of guilt or disappointment when their infant was unable to complete the protocol, despite recognizing that such outcomes were common and outside of their control. Consistent with prior reports identifying infant sleep and parental anxiety as key challenges in pediatric neuroimaging (Raschle et al., 2012b), our findings suggest that perceived responsibility for scan success may be a particularly important yet relatively underrecognized component of caregiver experience.

Importantly, caregivers offered several suggestions for mitigating these concerns. Participants emphasized the value of normalizing infant sleep unpredictability, clearly communicating that unsuccessful scan attempts are common, and reassuring families that participation remains meaningful regardless of the amount of data acquired. While pre-scan sleep questionnaires may be helpful for scheduling scans around typical naptimes, they should be used with caution given caregiver feedback that being asked about nap schedules can trigger feelings of anxiety when their infant does not have a predictable nap schedule. Thus, balancing family scheduling preferences while normalizing the unpredictability of infant sleep is critical. Caregivers’ comments also suggest the importance of acknowledging the emotional effort and sense of responsibility that many families bring to the research process. Although some caregivers noted that no amount of reassurance could fully eliminate the pressure they placed on themselves, these findings suggest that emphasizing that scan outcomes are not a reflection of caregiver actions or effort may help reduce anxiety during participation.

### 4.4 Experience and familiarity across visits

Caregivers consistently described becoming more comfortable with the MRI process over time. Initial uncertainty and hesitation were most apparent prior to the first visit, when families were more unfamiliar with the scanner environment and study procedures. However, after observing the procedures firsthand and successfully navigating a visit, many caregivers reported feeling substantially more prepared and confident during subsequent visits.

These findings are consistent with previous work suggesting that familiarity with MRI procedures can reduce anxiety and improve participation experiences (Raschle et al., 2012b; Dean et al., 2013). Importantly, caregivers in the present study received extensive information regarding MRI safety and study procedures prior to participation, suggesting that increased comfort across visits was not simply a consequence of greater knowledge. Rather, firsthand experience with the scanner environment, study procedures, and interactions with research staff appeared to reduce uncertainty in ways that were difficult to achieve through written or verbal explanations alone. Consequently, efforts to make the first visit feel more like later visits may be particularly valuable. Several caregivers proposed pre-visit materials, including photographs, websites, or brief videos introducing the scanner environment, equipment, study procedures, and visit flow. Such materials may be useful not because they provide additional information, but because they help approximate aspects of the first-visit experience prior to participation, thereby increasing familiarity, reducing uncertainty, and improving preparedness.

### 4.5 The role of communication in shaping caregiver experiences

Communication emerged as a central theme throughout the focus groups and appeared to shape caregiver experiences before, during, and after study visits. Caregivers consistently emphasized the importance of clear communication throughout the research process, including scheduling and study procedures, what to expect before and during scanning (e.g., scanner sounds, scan duration, and communication once their infant had fallen asleep), opportunities for repeat scans, and what constituted a successful scan. Across focus groups, caregivers described supportive communication from the research team as a key factor in alleviating concerns, particularly those related to infant sleep and scan completion.

Importantly, caregivers highlighted that effective communication involved not only what information was communicated, but also how and when information was shared. Several participants noted that procedural information delivered during the visit was difficult to absorb while simultaneously attempting to feed, rock, or settle their infant. Consequently, caregivers preferred receiving detailed procedural information before the visit, when they were better able to review and process it.

Beyond conveying information, communication helped build rapport throughout participation. Raschle et al. (2012a) emphasized that caregiver comfort can directly influence infants’ feelings of safety and well-being, a particularly important consideration for MRI studies conducted during natural sleep. In the present study, caregivers repeatedly described supportive interactions with research staff—such as normalizing infant sleep challenges, clearly explaining each step of the protocol, and providing reassurance when scans were unsuccessful—as critical to their overall experience. Caregivers also identified opportunities for improving communication during visits, including more explicit updates regarding scan progress and elapsed time, as well as quieter methods of communicating with research staff once their infant had fallen asleep. In addition, caregivers valued a collaborative approach in which research staff were responsive to both infant and caregiver cues when determining whether to continue or stop a scan. Together, these findings suggest that effective communication extends beyond simply providing information about study procedures and instead requires thoughtful consideration of when information is shared, ongoing communication with families throughout participation, and responsiveness to caregiver and infant cues.

### 4.6 Integrating caregiver perspectives into infant neuroimaging research

The present study extends prior work examining caregiver attitudes toward participation in infant MRI research (Kohlasch et al., 2021) by qualitatively examining caregivers’ lived experiences following participation in natural-sleep infant MRI research. An important outcome of the present study was that caregiver feedback directly informed subsequent changes to our infant MRI procedures. For example, we added a clock visible from the scanner room so caregivers could track elapsed time and introduced use of a squeeze ball to allow caregivers to notify research staff when their infant had fallen asleep or when assistance was needed without knocking on or opening the scanner-room door. To help reduce anxiety surrounding scan success, we now inform families in advance that unsuccessful scan attempts are expected and that there may be opportunities to reattempt the scan. We have also developed a study website with video content to increase families’ familiarity with the MRI environment and procedures before their visit. In response to requests for greater scheduling flexibility, we began offering Saturday MRI appointments. These examples illustrate how caregiver input can translate directly into changes to research procedures.

Although caregiver input in the present study was obtained after the MRI protocol had already been developed, our experience suggests that engaging caregivers prospectively during protocol development may help identify potential challenges and refine procedures before data collection begins. Participatory research frameworks increasingly advocate for involving participants and families early and throughout the research process (Sheridan et al., 2017), yet such early engagement remains relatively uncommon. For example, a review of parents as co-researchers found that parent involvement was largely absent during study conceptualization, despite evidence that their involvement increased the relevance of research to the target population and identified practical issues not anticipated by researchers (Shen et al., 2016). Similarly, a recent review of patient and family engagement in child health research found positive impacts on the research process in nearly all included studies and concluded that engagement can strengthen research relevance and feasibility while benefiting both research teams and patient and family partners (Vanderhout et al., 2023). In infant neuroimaging, where study procedures depend heavily on caregiver participation, engaging caregivers not only when evaluating and refining existing protocols, but also prospectively when designing infant MRI research procedures, may be particularly valuable.

### 4.7 Limitations

Several limitations should be considered when interpreting findings from the present study. First, our sample consisted of caregivers who enrolled in the MRI portion of the parent study and thus may represent caregivers who were more comfortable with research participation or who had more positive experiences than families who declined participation or withdrew from the study. Future studies may benefit from a broader range of caregiver perspectives, such as families who withdrew from participation or who chose not to enroll in infant MRI research during the initial recruitment process. Second, while the focus group participants were generally representative of the parent study cohort, the sample was predominantly White and of relatively high socioeconomic status and therefore may not reflect the diversity of families in the broader metropolitan Atlanta population or other populations participating in infant neuroimaging research. Third, individual speakers could not be reliably linked to participant characteristics across all three focus groups, which limited our ability to examine whether motivations for participation, experiences, or recommendations differed according to familial autism likelihood status or prior MRI scan success. Although our purposive recruitment strategy ensured representation across these characteristics, we cannot determine whether the themes identified here were experienced similarly across these groups. Finally, although the focus group format generated informative discussions, it may also have introduced social desirability bias or limited participants’ willingness to share dissenting or negative experiences in a group setting. In addition, the small number of participants in some focus groups may have limited opportunities for interaction and exchange among caregivers, although the smaller group format may have provided greater opportunity for individual participants to elaborate on their experiences and for the moderator to explore these experiences in depth.

## 5 Conclusion

Although participants generally described positive experiences, the focus groups revealed an unanticipated source of anxiety centered on infant sleep and caregivers’ sense of responsibility for scan success, rather than the MRI procedure itself. Caregivers provided recommendations for improving study procedures, including increasing opportunities for familiarity prior to the first visit, normalizing challenges associated with infant sleep, and fostering collaborative communication throughout participation. These findings demonstrate how partnering with participating families can identify unanticipated challenges and generate practical, caregiver-informed strategies for advancing more family-centered and effective infant neuroimaging research.

## Data Availability

The raw data supporting the conclusions of this article will be made available by the authors, without undue reservation.
