# Supplementary material for: Caregiver perspectives on participation in pediatric neuroimaging research: insights from qualitative focus groups
Source: Front Neurosci. 2026 Oct 14;20:1963995. doi: 10.3389/fnins.2026.1963995 (PMC13713826; doi:10.3389/fnins.2026.1963995)
Supplement: Supplementary file 1 [file Supplementary_file_1.docx]

Appendix A.

*Semi-Structured Interview Guide for Caregiver Focus Groups*

This interview guide was used during caregiver focus groups to assess motivations for participation, experiences with the infant MRI study, and suggestions for future protocol improvements.

1. Overall experience

- How was your experience participating in the MRI study?
- Is there anything about your experience that you’d like to share?

2. Pre-scan process

- How did the eligibility and scheduling process feel?
- What was your experience with the naptime questionnaire?
- Was the information provided before the scan helpful?
- How did you feel about scheduling flexibility?

3. Scan room impressions

- What were your first impressions of the MRI suite and scanning room?
- Was the level of detail provided before entering sufficient?

4. Infant sleep and scan preparation

- What helped or hindered your infant in falling asleep?
- How did you feel being alone in the room with your baby?
- What was your experience transitioning your baby to the scanner bed?

5. During the scan

- How did you feel watching your baby through the camera?
- Did your baby stir or wake up, and how did that affect your experience?
- How did you feel about the scan duration?
- Would a visible clock or updates from staff helped?
- Was the stopping/starting of the scanner clearly explained?
- Did you feel you could communicate effectively with the team?

6. Research “brain diploma”

- How did you feel about receiving brain images as a keepsake?

7. Post-scan experience

- How did you feel when the scan ended?
- Was follow-up scheduling clear and convenient?

8. Longitudinal participation

- How did later visits compare to your first visit?

9. Recommendations and reflections

- What worked well?
- What should we improve?
- Did anything go wrong or feel unclear?
- Would you participate again or recommend this study to others?
- Do you feel your participation contributed to science?

10. Prior research experience

- Have you participated in other research?
- How did this experience compare?

Appendix B.

*Examples of Thematic Coding Summary for “Suggestions”*

| Subtheme | Description | Focus Group (FG) |
| --- | --- | --- |
| Flexibility in scheduling | Caregivers suggested providing an option to come back later in the week if their baby was unable to sleep. | FG1, FG2, FG3 |
| Time parameters | Request for a clock around the scan room to keep track of time duration. | FG1, FG2, FG3 |
| Informational MRI video | A short informational clip sent to caregivers prior to study visits to explain MRI study procedures and safety. | FG2, FG3 |
